# A Naturally Occurring Lgr4 Splice Variant Encodes a Soluble Antagonist Useful for Demonstrating the Gonadal Roles of Lgr4 in Mammals

**DOI:** 10.1371/journal.pone.0106804

**Published:** 2014-09-04

**Authors:** Pei-Jen Hsu, Fang-Ju Wu, Masataka Kudo, Chih-Lun Hsiao, Aaron J. W. Hsueh, Ching-Wei Luo

**Affiliations:** 1 Department of Life Sciences and Institute of Genome Sciences, National Yang-Ming University, Taipei, Taiwan; 2 Division of Reproductive and Stem Cell Biology, Department of Obstetrics and Gynecology, Stanford University School of Medicine, Stanford, California, United States of America; Baylor College of Medicine, United States of America

## Abstract

Leucine-rich repeat containing G protein-coupled receptor 4 (LGR4) promotes the Wnt signaling through interaction with R-spondins or norrin. Using PCR amplification from rat ovarian cDNAs, we identified a naturally occurring *Lgr4* splice variant encoding only the ectodomain of Lgr4, which was named Lgr4-ED. Lgr4-ED can be detected as a secreted protein in the extracts from rodent and bovine postnatal gonads, suggesting conservation of Lgr4-ED in mammals. Recombinant Lgr4-ED purified from the conditioned media of transfected 293T cells was found to dose-dependently inhibit the LGR4-mediated Wnt signaling induced by RSPO2 or norrin, suggesting that it is capable of ligand absorption and could have a potential role as an antagonist. Intraperitoneal injection of purified recombinant Lgr4-ED into newborn mice was found to significantly decrease the testicular expression of estrogen receptor alpha and aquaporin 1, which is similar to the phenotype found in *Lgr4*-null mice. Administration of recombinant Lgr4-ED to superovulated female rats can also decrease the expression of estrogen receptor alpha, aquaporin 1, LH receptor and other key steroidogenic genes as well as bring about the suppression of progesterone production. Thus, these findings suggest that endogenously expressed Lgr4-ED may act as an antagonist molecule and help to fine-tune the R-spondin/norrin-mediated Lgr4-Wnt signaling during gonadal development.

## Introduction

Genomic studies of leucine-rich repeat containing G protein-coupled receptors (LGRs) from diverse species have indicated that LGRs can be subdivided into three groups [1], [2]. The ligands for the group A and group C LGRs are glycoprotein hormones and relaxin/insulin-like peptides, respectively. Intriguingly, several studies have demonstrated that the group B LGRs are able to interact with R-spondins [3], [4], whereas our recent study has shown that they are also able to bind to bursicon-like molecules such as bursicon and norrin [5], [6].

The mammalian group B LGRs have recently gained prominence as potential stem cell markers and they seem to play crucial roles in maintaining stem cell functions in diverse tissues. For example, LGR4 is required for maintenance of stem cells in the intestine, mammary gland and prostate [7]–[9]. LGR5 is a marker of stem cells located in the crypts of the gastrointestinal tracts [10], [11], the nascent nephrons of the kidney [12], and the hair follicles [13]. LGR6-positive stem cells in the hair follicles have been found to be capable of generating all cell lineages of the skin [14].

In addition to their vital roles in stem cells, studies using mutant animal models have also shown that the group B LGRs are essential during mammalian development. For example, it has been demonstrated that the *Lgr4* gene displays a very wide expression, with stronger signals being present in the kidney, adrenal gland, bone/cartilage, gastrointestinal tracts, heart, reproductive tracts and nervous systems [15], [16]. As a result of this wide distribution, the phenotypes of *Lgr4*-null mice are quite complicated. Disruption of the *Lgr4* gene in mice on the C57B1/6J x Swiss Webster background led to perinatal lethality and intrauterine growth retardation; these effects were associated with pronounced decreases in the weights of the kidney and liver [16]. In contrast, *Lgr4*-null mice that have a CD1 background are viable; nevertheless, male *Lgr4*-null mice are sterile and have a number of major defects affecting their reproductive tracts; these include the dilated *rete testis* and absence of sperms in the epididymis [17]. Further studies have suggested that Lgr4 plays pivotal roles in regulating the expression of estrogen receptor alpha (*Esr1*) and aquaporin (*Aqp1*), remodeling of basement membrane, and regional differentiation of the male reproductive tracts via epithelial-mesenchymal interactions [17], [18].

In addition to the testis, the *Lgr4* gene is also abundantly expressed in ovarian follicles and has an even higher expression level in corpus luteum [15]. However, the expression profiles of *Lgr4* in the postnatal gonads as well as how the Lgr4 signaling affects postnatal ovarian development have not yet been well characterized. In the present study, we identified a naturally occurring *Lgr4* splice variant encoding only the extracellular ectodomain of Lgr4 and named it as Lgr4-ED. The recombinant Lgr4-ED protein was further generated to characterize its potential roles in the postnatal testis and ovary.

## Materials and Methods

### Ethics statement

All animals were housed under a controlled humidity, temperature, and light regimen in strict accordance with the recommendations in the Guide for the Care and Use of Laboratory Animals of the National Yang-Ming University. Animal treatments and sacrifice were approved by the Institutional Animal Care and Use Committee of the National Yang-Ming University (Permit Number: 1021229). All efforts were made to minimize animal suffering.

### Animal treatments and progesterone assays

C57B1/6J mice and Sprague-Dawley rats were obtained from the laboratory animal center (National Yang-Ming University, Taipei, Taiwan) and randomly allocated into groups. For testing the Lgr4-ED effects on mouse testes, neonatal male mice were intraperitoneally injected with 10 µg of the purified Lgr4-ED protein every week and the testes were harvested on day 22. For time-course analyses of *Lgr4* expression in the superovulation model, immature female rats (26-day old) were primed with 15 IU pregnant mare serum gonadotropin (PMSG) at 0900–1000 A.M. and received an intraperitoneal injection of 10 IU human chorionic gonadotropin (hCG) 48 h later. For Lgr4-ED injection in the superovulation model, 20 µg of the purified Lgr4-ED protein was co-injected with PMSG intraperitoneally and the ovaries were harvested at 48 h after injection.

For analyzing the changes of progesterone, the harvested ovaries were weighed and homogenized in cold PBS. The protein contents in collected supernatants were measured for normalization using the Micro BCA protein assay kit (Pierce Biotechnology). The amounts of progesterone in the supernatants were measured by ELISA using the specific anti-progesterone antibody (Sigma).

### Cloning of the *Lgr4* splice variant and generation of the recombinant Lgr4-ED protein

The *Lgr4* splice variant was identified during PCR amplification of *Lgr4* from the mature rat ovarian cDNA by Dr. Masataka Kudo using primers 5-TTGGAGAGTCTAACCTTG-3 and 5-TTAATAGCACTAAGGTCACAG-3. The cDNA construct encoding the Lgr4-ED protein was kindly provided by Dr. Aaron Hsueh at Stanford University. To facilitate recombinant protein purification, the construct was designed by adding a FLAG epitope tag at the N-terminus. The constructed plasmid was purified and then transfected into human 293T cells using LipofectAMINE 2000 (Life Technologies). Transfected cells were selected by the Zeocin-containing medium. The selected cells were allowed to reach confluence and then cultured for 72 h in the serum-free medium. Conditioned media were collected, filtrated and then subjected to anti-FLAG M1 affinity gel (Sigma) for protein purification. Measurement of the protein content was carried out by Micro BCA protein assay kit (Pierce Biotechnology). The purity and biochemical characteristics of the purified protein were analyzed by electrophoresis on a 10% SDS polyacrylamide gel.

### cDNA isolation and real-time PCR quantification

For cDNA preparation, the total RNAs of harvested tissues or cells were extracted using the TRIzol reagent according to manufacturer instructions (Life Technologies). Five micrograms of total RNA were then reverse-transcribed using RevertAid First Strand cDNA Synthesis kit (Fermentas, Hanover, MD) with the oligo-dT primer. For quantitative TaqMan real-time PCR, a QuantiTect Probe PCR kit (QIAGEN Sciences) was used. The primer pairs and fluorescent probes for each gene were listed as follows: Rat *Lgr4* forward, CAGTGCCCAGTGAAGCCATTC; rat *Lgr4* reverse, TGTTGTCATCCAGCCACAGA. Mouse *Lgr4* forward, CAGTACCCAGTGAAGCCATTC; rat *Lgr4* reverse, TGTTGTCATCCAGCCACAGA; rat and mouse *Lgr4* probe, TAGATGCCAACCATATTACCTCAGTCC. Rat and mouse *Actb* forward, CTCTGTGTGGATTGGTGGCTC; rat and mouse *Actb* reverse CTGCTTGCTGATCCACATCTG; rat and mouse *Actb* probe CCTGGCCTCACTGTCCACCTTCC. Rat *Lhr* forward, GTATCAGCAATTACATGAAGGTCAG; rat *Lhr* reverse, AGATCCTAATGTAGCAAGCACAGAT; rat *Lhr* probe, CTTAATCCTCAACGTGGTGGCCTTC. Rat *Star* forward, AGATGAAGTGCTAAGTAAGGTGGTG; rat *Star* reverse, CCAGTTCTTCATAGAGTCTGTCCAT; rat *Star* probe, TCTAGCAGCACCTCCAGTCGGAACA. Rat *Hsd3b* forward, AGACCATCCTAGATGTCAA-TCTGAA; rat *Hsd3b* reverse, CAGGATGATCTTCTTGTAGGAGT; rat *Hsd3b* probe, TCTACTGCAGCACAGTTGACGTTGC. Rat *Cyp11a1* forward, CCAAGTTCAACCTCATCCTGA; rat *Cyp11a1* reverse, GTGTGACTGCAGCCTGCAAT; rat *Cyp11a1* probe,CTTCAACTTCCAGCCTCTCAAGCAG.

For the quantitative SYBR Green real-time PCR, SYBR Green PCR Master Mix kit (Life Technologies) was used and the primer pairs for each gene were listed as follows: Rat and mouse *Esr1* forward, GCTGCGCAAGTGTTACGAA; rat and mouse *Esr1* reverse, CATTTCGGCCTTCCAAGTCAT. Rat and mouse *Esr2* forward, GAGGCAGAAAGTAGCCGGAA; rat and mouse *Esr2* reverse, CGTGAGAAAGAAGCATCAGGA. Rat *Aqp1* forward, ATCACCTCCTCCCTGCTCGA; rat *Aqp1* reverse, AGTGTAGTCAATGGCCAGCA. Mouse *Aqp1* forward, ATCACCTCCTCCCTAGTCGA; mouse *Aqp1* reverse, AGTGTAGTCAATCGCCAGCA. Rat and mouse *Actb* forward, CTCTGTGTGGATTGGTGGCTC; rat and mouse *Actb* reverse TTGCGCAAGTTAGGTTTTGTC.

### Immunoblotting and immunohistochemical analyses

The rabbit anti-Esr1 antibody was purchased from Santa Cruz Biotechnology. The mouse anti-FLAG monoclonal antibody was from Sigma-Aldrich Corp. The rabbit anti-Lgr4 antibodies were from Abcam and Dr. Aaron Hsueh. To extract full-length Lgr4 from transfected 293T cells, the cells were lysed in ice-cold RIPA lysis buffer (50 mM Tris-HCl, pH 7.4, 150 mM NaCl, 1 mM dithiothreitol, 1% NP40, 0.1% SDS) supplemented with protease inhibitor cocktails (Roche). The lysate was pelleted by centrifugation at 16,000 g for 15 min at 4°C and the supernatant was mixed with 5X sample buffer and incubated at 37°C for 1 hr before analyzed by SDS-PAGE. The bovine follicular fluid was collected from a local slaughterhouse. Rat ovaries and mouse testes were homogenized in ice-cold PBS. Following centrifugation, protein amounts in the supernatants were quantified by Micro BCA protein assay kit (Pierce Biotechnology) before subjecting to electrophoresis and immunoblotting assays.

For immunohistochemical analyses, the ovaries and testes harvested from treated animals were fixed in Bouin’s fixative and embedded. Tissue sections were probed with specific primary antibodies. Substitution for the primary antibodies with the rabbit preimmune serum or normal IgG served as the negative controls. Staining was visualized using the HRP-conjugated secondary antibody followed by the Nova Red kit (DakoCytomation) or Alexa Fluor 488-conjugated secondary antibody (Life Technologies) followed by observation under a fluorescent microscope.

### Wnt reporter luciferase assays

TOPFLASH–luciferase assays were performed by transfecting human LGR4 or LGR4 and human norrin plasmids as described previously [5]. Briefly, for RSPO2 treatment, 293T cells were seeded into 24-well plates and transfected with human LGR4 (0.05 µg) and pCMV-β-Gal (0.01 µg) plasmids using LipofectAMINE 2000 (Life Technologies). At 24 h after transfection, cells were further treated with or without WNT3A (1 nM) and RSPO2 (3 nM) for another 16 h in serum-free medium. For norrin expression, 293T cells were transfected with LGR4 (0.05 µg), pCMV-β-Gal (0.01 µg) and/or increasing amounts of norrin plasmid using LipofectAMINE 2000 (Life Technologies). At 24 h after transfection, cells were further cultured in serum-free medium for another 16 h. Luciferase activities were determined using luciferase assay kits (Promega) and normalized using β-galactosidase activities. All experiments were performed at least three times in triplicate.

### Data analysis

For comparisons of significant difference, the values were subjected to analysis by the Student’s *t*-test. Significance was accepted at *P<*0.05 and is indicated by asterisks in the figures unless noted otherwise.

## Results

### Identification of a *Lgr4* splice variant encoding only the ectodomain of *Lgr4* protein

**Figure 1:**
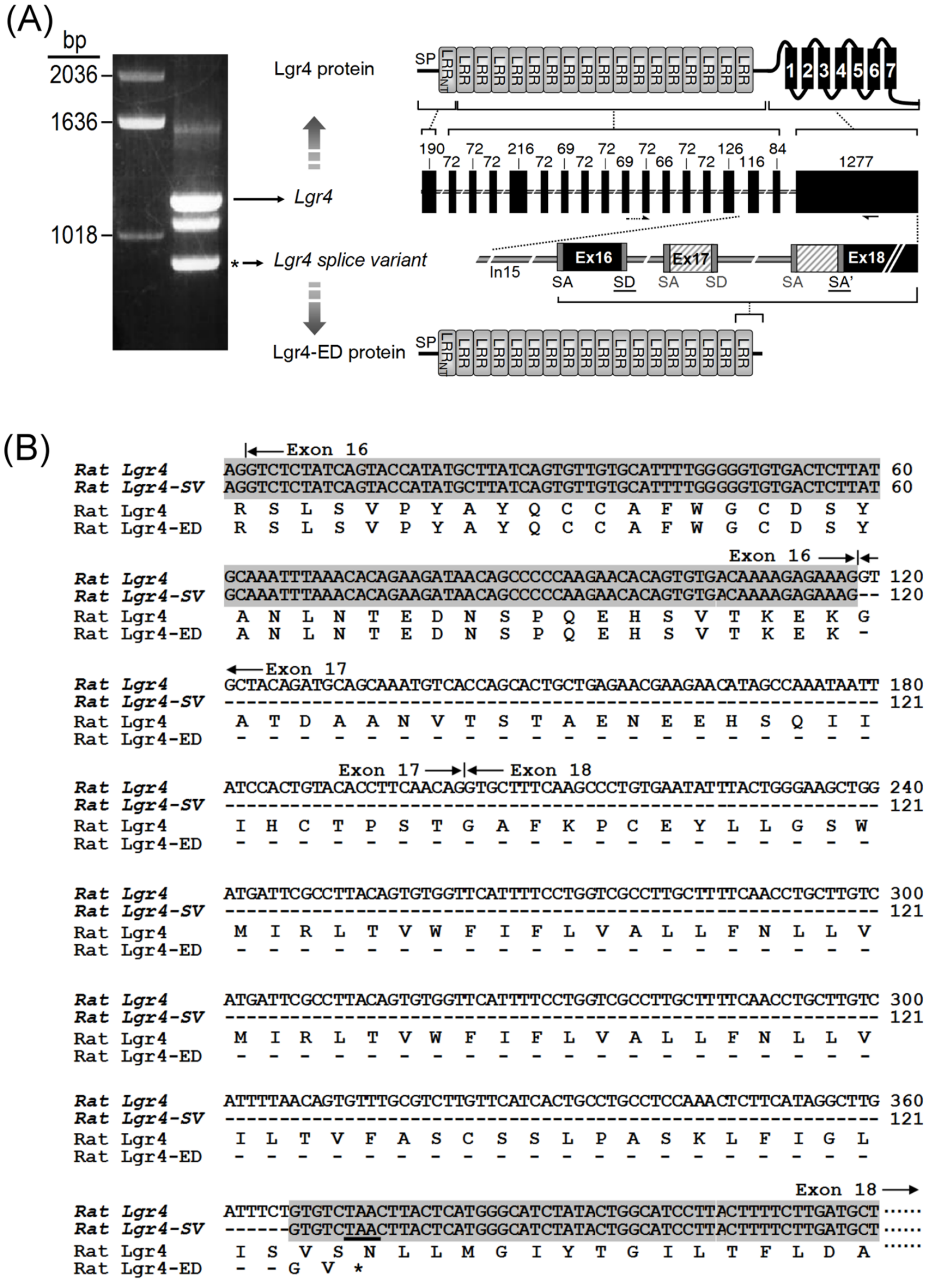
Gene structure of *Lgr4* and the amino acid sequence of Lgr4-ED. (A) Cloning and gene organization of rat *Lgr4* and rat *Lgr4* splice variant. Left panel: both fragments of full-length *Lgr4* and *Lgr4* variant can be amplified from the mature rat ovarian cDNA by using a forward primer spanning exon 10 and 11 and a reverse primer in exon 18. Right panel: exons are represented by *boxes*; introns are shown by the *lines* between exons. The numbers indicate the base pairs in each exon. Primer locations were indicated by *arrows* at the bottom of axons. The *Lgr4* variant is spliced via selection of the splice donor (SD) site of exon 16 and an alternative splice acceptor (SA’) of exon 18; this results in the deletion of exon 17 and the initial 164 bps of exon 18 (slash boxes). The deduced domain structure from each exon of the full-length Lgr4 and Lgr4-ED proteins are shown. SP, signal peptide; LRRnt; leucine rich repeat N-terminal domain; LRR; leucine rich repeat domain; TM, transmembrane domain. (B) Comparison of nucleotide sequences and deduced amino acids between rat *Lgr4* and rat *Lgr4* splice variant (*SV*). *Dashed lines* indicate gaps introduced in the sequence for optimal alignment.

During PCR amplification of the *Lgr4* fragment from mature rat ovaries, a previously uncharacterized splice form was found (Fig. 1A, left panel). The splice variant was then cloned and sequenced. Genomic structure analysis indicated that this splice form consists of the first 16 exons of *Lgr4* (Fig. 1A, right panel). Sequencing data indicated that alternative splicing had resulted in deletion of exon 17 and recreation of a previously unidentified exon/intron junction in exon 18 of the *Lgr4* gene, thus leading to a frame-shift and the introduction of an early termination codon before the translation of seven-transmembrane domains of Lgr4 (Fig. 1B). Consequently, this splice variant encodes the first 500 amino acids of Lgr4, including the signal peptide, N-flanking cysteine-rich and leucine-rich repeat sequence, 17 leucine-rich repeat motifs and C-flanking cysteine-rich sequence (Fig. 1A, right panel). The resulting protein contains only the extracellular portion of Lgr4 and thus was named Lgr4 ectodomain, Lgr4-ED.

**Figure 2:**
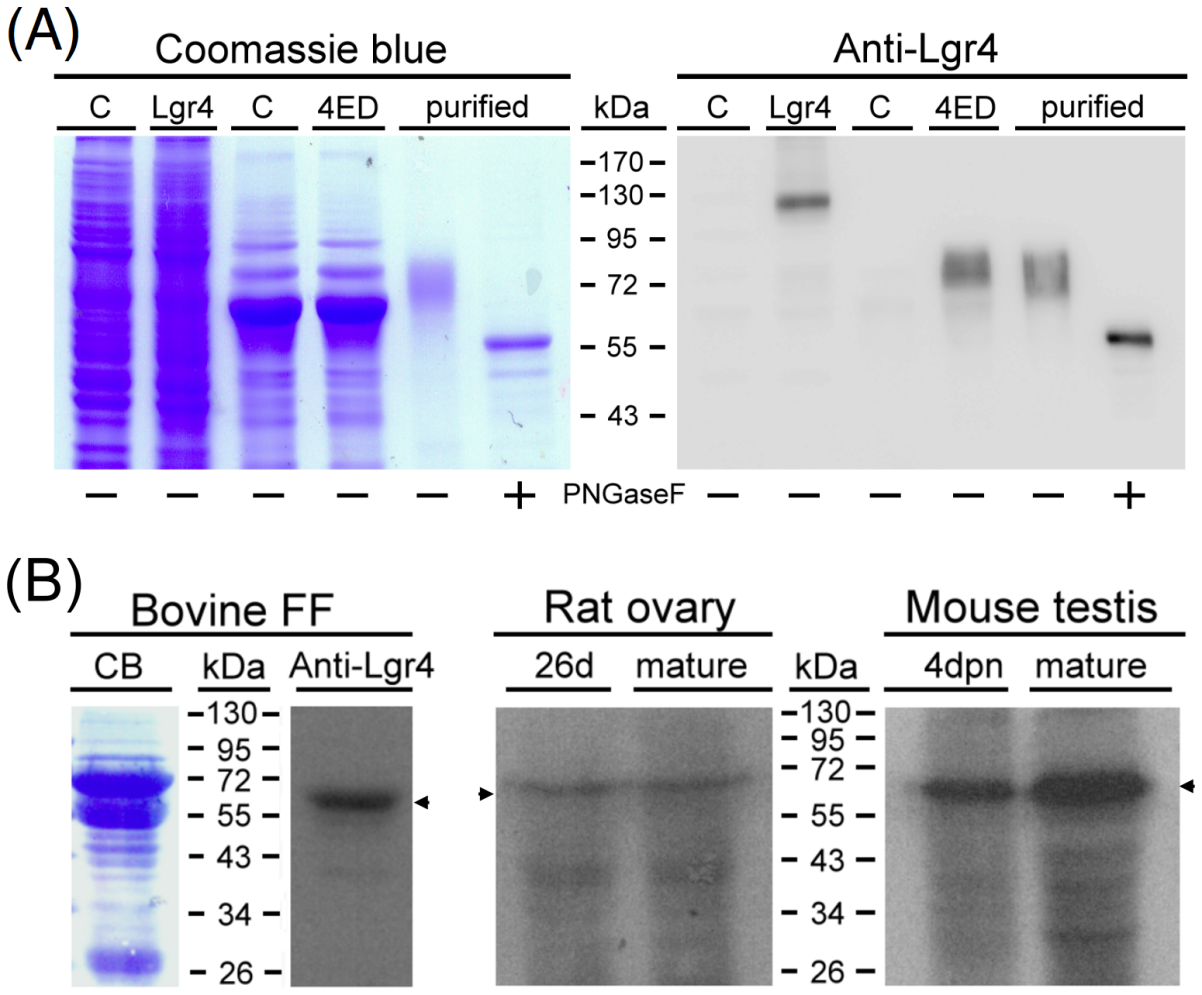
Recombinant and endogenous expression of the Lgr4-ED proteins. (A) SDS-PAGE analysis of full-length rat Lgr4 in transfected 293T cells and recombinant N-terminal FLAG-tagged rat Lgr4-ED (4ED) secreted by transfected 293T cells. C: cells transfected with control pcDNA3.1. Purified Lgr4-ED was further treated without or with PNGase F to characterize its glycosylated nature. The gels were stained using Coomassie blue (left panel) or probed with the anti-Lgr4 antibody (right panel). To adjust the band signals, 1.5 µg and 0.015 µg of purified Lgr4-ED before or after de-glycosylation was loaded for Coomassie blue staining and Western blotting, respectively. (B) Detection of the endogenous Lgr4-ED protein signals in different species. Bovine follicular fluid (FF) and the homogenized supernatants from rat ovaries and mouse testes were subjected to SDS-PAGE and Western analysis against Lgr4. CB, Coomassie blue staining; dpn, days postnatal.

Lgr4-ED was predicted to be a secreted protein due to the lack of seven-transmembrane domains of the original protein. To confirm this, the cDNA corresponding to full-length rat Lgr4 or Lgr4-ED was constructed into pcDNA3.1 to allow mammalian cell expression. Western blot analyses indicated that the Lgr4 antibody can specifically recognize the full-length Lgr4 protein migrated as a band around 130 kDa. The recombinant Lgr4-ED protein was capable of being secreted from transfected 293T cells and had a molecular weight around 70 kDa (Fig. 2A, right panel). We further purified the recombinant FLAG-tagged Lgr4-ED protein from the conditioned media using affinity chromatography. Under reducing conditions, purified Lgr4-ED migrated as a band around 70 kDa (Fig. 2A, left panel, lane 5). The protein migrated faster after treatment with N-glycosidase F (Fig. 2A, left panel, lane 6), indicating the N-linked glycosylation of the recombinant Lgr4-ED protein.

To further confirm the endogenous translation of Lgr4-ED, protein samples from the gonads of various species, including the follicular fluid collected from bovine ovaries and the supernatants from tissue homogenates of rat ovaries and mouse testes at different ages, were used (Fig. 2B). Western analysis using the anti-Lgr4 antibody showed a specific band around 70 kDa in the bovine follicular fluid. This signal did not overlap with prominent albumin bands as compared to the protein migration positions visualized by Coomassie blue staining, suggesting that the Lgr4 antibody was specific. In homogenates prepared from immature or mature rat ovaries and immature or mature mouse testes, signals of a similar molecular size were also detected. Taken together, these findings suggest that the Lgr4-ED protein is naturally secreted by both types of gonads of various mammals.

### Functional tests of Lgr4-ED

**Figure 3:**
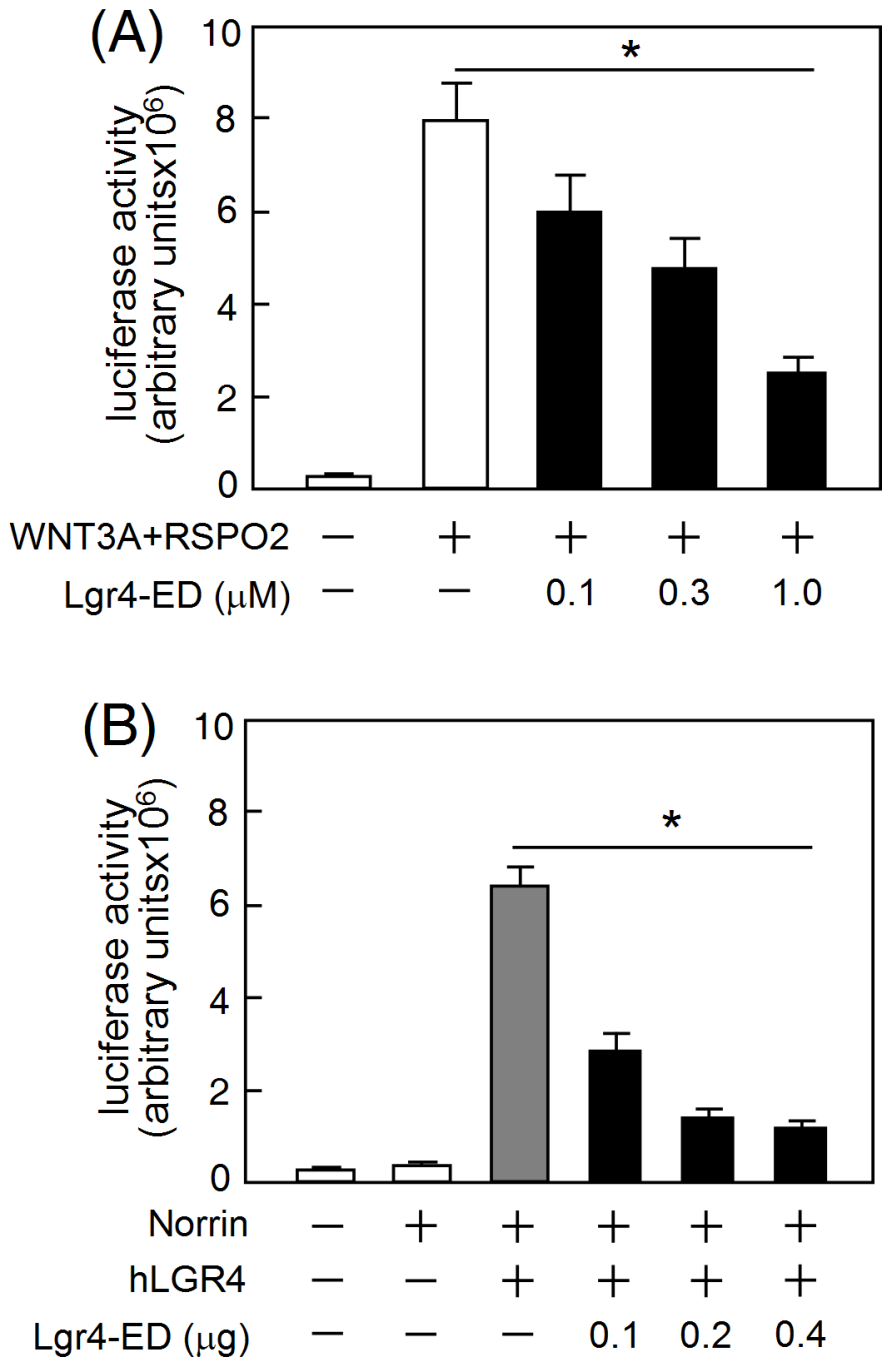
Antagonist effects of Lgr4-ED. (A) For the R-spondin assays, HEK293T cells were transfected with plasmids encoding TOPFLASH, CMV-β-Gal and human LGR4. At 24 h after transfection, cells were further treated with WNT3A, RSPO2 and graded amounts of purified Lgr4-ED as indicated. (B) For the norrin assays, HEK293T cells were co-transfected with TOPFLASH, CMV-β-Gal, LGR4, norrin and increasing amounts of rat Lgr4-ED plasmids as indicated. After recovery in serum-containing medium for 24 h, the cells were incubated in the serum-free medium for 16 h before performing luciferase assays. Luciferase data were normalized against the β-galactosidase levels and are shown as the mean ± SD of triplicates (*, *P*<0.05). Three additional experiments were performed and all three produced similar results.

We further evaluated the bioactivity of the recombinant Lgr4-ED protein. Lgr4 has been demonstrated to be a receptor for R-spondins that mediate the Wnt signal enhancement [3], [4]. The combination of WNT3A with RSPO2 led to a significant increase of the Wnt signaling as measured by the TOPFLASH reporter assay. In contrast, exogenous addition of the recombinant Lgr4-ED suppressed this response in a concentration-dependent manner (Fig. 3A). In addition, norrin is also able to stimulate the Wnt signaling mediated by human LGR4 [5], whereas such a stimulation can be reversed by co-expression of Lgr4-ED (Fig. 3B). These findings suggest that the recombinant Lgr4-ED protein acts as a functional antagonist capable of neutralizing the Wnt signaling induced by R-spondins and norrin.

### Testicular and ovarian expression of *Lgr4*

**Figure 4:**
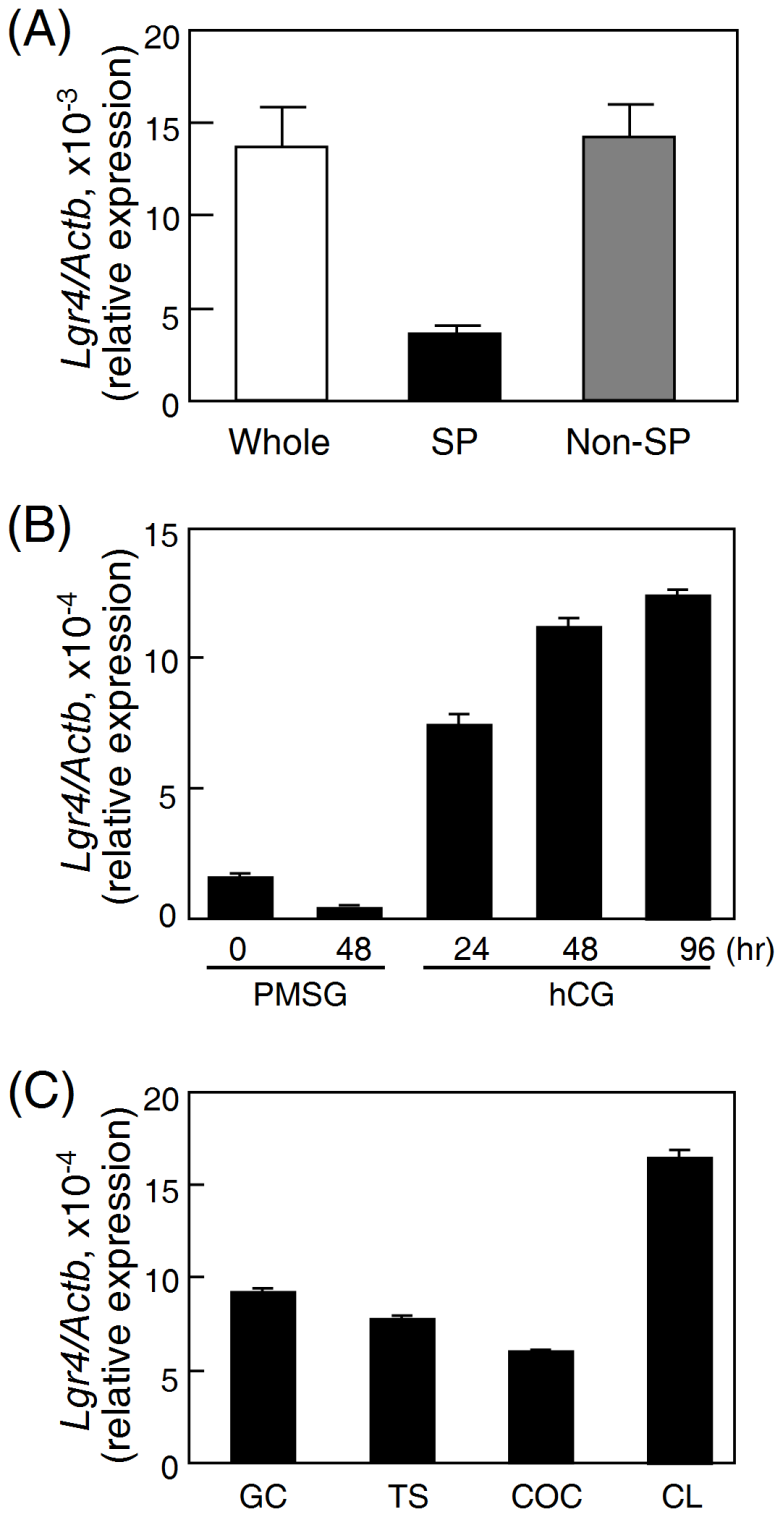
Expression of *Lgr4* in the postnatal gonads. (A) Comparison of *Lgr4* expression in different testicular cell types. The whole testes from mice at 7 days postnatal and the isolated spermatogonia (SP) and non-spermatogonia (non-SP) were used for real-time quantification of the *Lgr4* expression levels. (B) Female rats at 26 d of age were treated with PMSG, followed 48 h later by hCG for different intervals. The ovaries at indicated intervals were harvested for real-time quantification of the *Lgr4* expression level. (C) Comparison of *Lgr4* expression in different ovarian cell types by real-time PCR quantification. Granulosa cells (*GC*), theca shells (*TS*), and cumulus-oocyte complexes (*COC*) were isolated from rat ovaries (26 days old), whereas corpora lutea (*CL*) were obtained from PMSG-primed rats at 72 h after hCG treatment. Data were normalized using β-actin levels and are expressed as the mean ± S.D.

To determine the testicular cell types that express *Lgr4*, the testes from mice at 7 days of age were digested and the cell types were further separated using magnetic activated cell sorting (MACS) based on antibodies against Thy1 antigens expressed in spermatogonial stem cells [19]. As shown in Fig. 4A, the *Lgr4* mRNA is mainly detected in the Thy1^−^ somatic cell-enriched fraction but not in the Thy1^+^ spermatogonial stem cell-enriched germ cell fraction.

To determine the expression of *Lgr4* in the ovary, the ovaries were collected from superovulated immature rats primed with PMSG followed by hCG for different intervals. Quantitative real-time PCR analyses showed that the *Lgr4* mRNA expression was elevated and remained at high levels 24 h after hCG injection (Fig. 4B). Subsequent analyses of the *Lgr4* transcript in various ovarian compartments, including granulosa cells, theca shells, cumulus-oocyte complexes and corpora lutea, indicated that *Lgr4* is widely expressed in these different ovarian cell types with corpora lutea showing the highest expression level (Fig. 4C).

**Figure 5:**
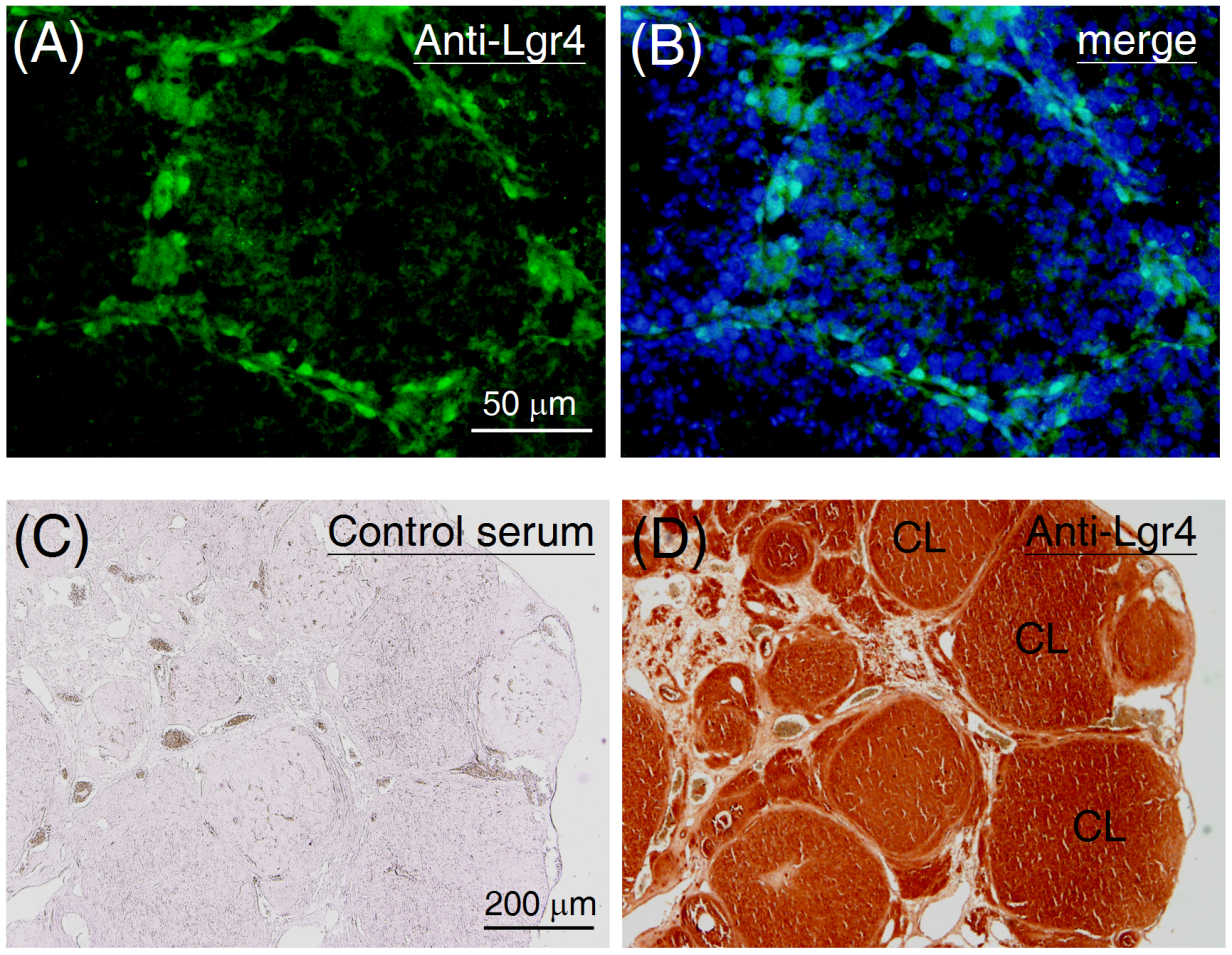
Distribution of the Lgr4 protein in the postnatal gonads. To assess the testicular distribution of Lgr4, testicular sections from mice at 5 weeks old were probed with the anti-Lgr4 antibody followed by fluorescein isothiocyanate-conjugated secondary antibody. Data are shown in parallel without (A) or with (B) a DAPI merged image. To assess the ovarian distribution of Lgr4, ovarian sections from mature rats (8 weeks old) were probed either (C) with preimmune rabbit serum or (D) with the anti-Lgr4 antibody. The sections were then visualized using a horseradish peroxidase system. CL, corpus luteum.

In addition, immunohistochemical analyses were also carried out to confirm the distribution of the Lgr4 protein. In mouse testes, the Lgr4 positive cells were mainly located around the periphery of the seminiferous tubules (Fig. 5A and 5B). Considering the *Lgr4* mRNA profile above and the cell morphology shown in the immunohistochemical staining, this data suggests that the Lgr4 protein is probably expressed in peritubular myoid cells but not in spermatogonia or Sertoli cells. In the ovaries harvested from adult rats (8 weeks old), a strong Lgr4 immunoreactivity was detected in corpora lutea (Fig. 5C and 5D), consistent with its mRNA profile.

### Lgr4-ED treatment decreases the expression of estrogen receptors and aquaporin 1 in the postnatal testis

**Figure 6:**
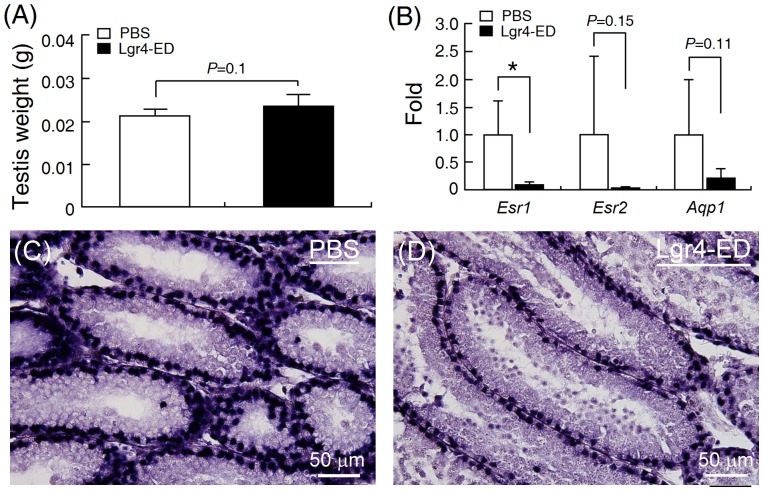
The recombinant Lgr4-ED protein affects postnatal development of the testis. Neonatal male mice were injected intraperitoneally with either controlled saline or purified recombinant Lgr4-ED (10 µg/animal) on postnatal day 1, 8 and 15. Mice were then sacrificed on day 22 and (A) the testicular weights were measured. (B) The expression levels of *Esr1, Esr2, Aqp1* in the harvested testes were compared by real-time PCR. Data were normalized against β-actin levels and represent the mean ± SEM (n = 6). *Asterisks* indicate *P*<0.05. (C and D) In addition, the testes were also subjected to immunohistochemical staining to detect the Esr1 protein and visualized using an alkaline phosphatase staining system.

It has been shown that *Lgr4* knockout leads to swelling and liquid accumulation in mouse testes as a consequence of down-regulation of the expression of estrogen receptor alpha (Esr1) and aquaporin (Apq1) [17], [18]. To test whether Lgr4-ED is a functional antagonist on testicular development *in vivo*, newborn male mice were intraperitoneally injected with Lgr4-ED on day 1, 8 and 15 after birth and their testes were harvested on day 22 for analyses. We found that Lgr4-ED administration did not affect the survival, body weights (controls: 8.4±1.3 g; treated: 8.5±1.4 g, n = 6) and testicular weights (Fig. 6A) of the treated mice. However, Lgr4-ED treatment was found to suppress the mRNA expression of estrogen receptors and *Aqp1*, with *Esr1* showing the most significant decrease (Fig. 6B). To confirm this, immunochemical staining against Esr1 was carried out and it was found that the Esr1 protein signal was significantly decreased in the testes of mice after Lgr4-ED injection (Figs. 6C and 6D). Taken together, our results suggest that Lgr4-ED administration can produce some of the phenotypes associated with *Lgr4*-knockout mice and this is likely via neutralizing the actions of endogenous Lgr4 ligands by Lgr4-ED.

### Lgr4-ED treatment suppresses ovarian development and steriodogenesis

**Figure 7:**
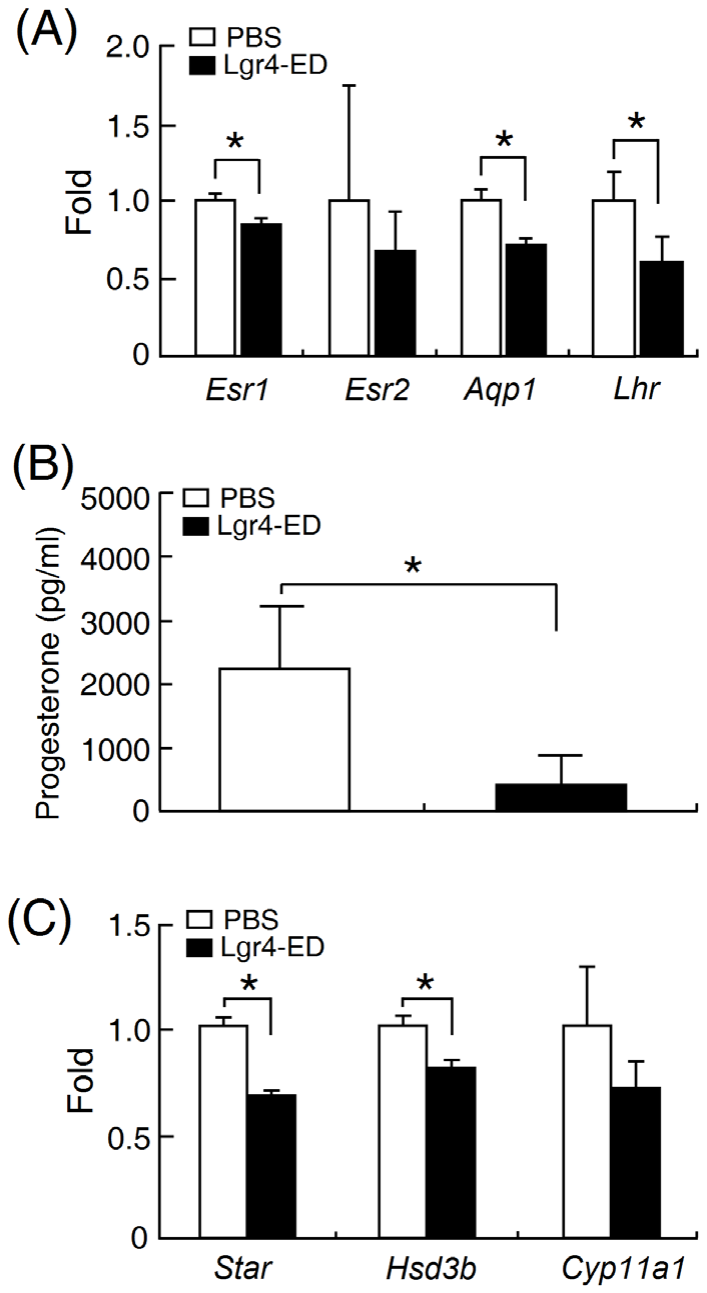
The recombinant Lgr4-ED protein decreases gonadotropin-driven ovarian development and steroidogenesis. Immature female rats (26 days old) were intraperitoneally injected with PMSG in the presence or absence of 20 µg Lgr4-ED. The ovaries were harvested two days later for assessing the expression levels of (A) *Esr1, Esr2, Aqp1* and *Lhr*. β-actin was used for normalization. (all bars; n = 7). In addition, (B) the progesterone amounts and (C) the transcript levels of various steroidogenic genes in the ovaries were evaluated (n = 4). Data are shown as the mean ± SEM. *Asterisks* indicate *P*<0.05.

To test the Lgr4-ED effects on ovarian development and steriodogenesis, immature female rats were intraperitoneally injected with PMSG together with or without the recombinant Lgr4-ED protein and their ovaries were then collected 2 days after injection. Although no significant changes in the ovarian weight (control: 0.031±0.0015 g; treated: 0.039±0.0041 g, n = 7) were detected, Lgr4-ED administration did significantly decrease the expression levels of *Esr1*, *Aqp1* and *Lhr* (Fig. 7A). In addition, Lgr4-ED injection also led to the suppression of PMSG-induced progesterone production (Fig. 7B) and this was accompanied by decreases in the transcript levels of various steroidogenic enzymes including steroidogenic acute regulatory protein and 3-β-hydroxysteroid dehydrogenase (Fig. 7C).

## Discussion

In the testis, undifferentiated spermatogonia are discontinuously scattered around the basement of seminiferous tubules. Although Lgr4 has been proposed as a stem cell marker in many tissues [7]–[9], our staining results showed that the Lgr4 signal is mainly located in the connected cells that form a continuous sheet surrounding the basement membrane of seminiferous tubules (Fig. 5A), suggesting that Lgr4 is unlikely to be located in spermatogonia. Real-time PCR quantification of isolated testicular cells further supported this hypothesis (Fig. 4A). Indeed, a previous study has also proposed that the *Lgr4* transcript is probably expressed in the myoepithelial cells of seminiferous tubules [15]. Using a gene trap approach, Qian *et al.* further demonstrated that the *Lgr4* transcript is selectively located in peritubular myoid cells but not in spermatogonia of postnatal mouse testes [20]; these findings are consistent with our results.

By disrupting the *Lgr4* gene with a trap vector carrying the β-galactosidase tracing enzyme, *Lgr4* in mouse ovaries has been proposed to be expressed in corpus lutea but not in other follicular compartments [15]. Although our results of mRNA quantification and protein staining in rat ovaries indicated that corpus lutea did express the highest level of *Lgr4*, we also detected moderate levels of the *Lgr4* transcript in other ovarian cells isolated from antral follicles (Fig. 4C). The stage difference between the appearance of *Lgr4* mRNA in rat ovaries and the appearance of translated β-galactosidase protein signal in mouse ovaries may be explained by the fact that different species were used or by effects of as yet uncharacterized regulatory factors that are induced after luteogenesis to control the initiation of translation of the *Lgr4* mRNA. In addition, sequence difference between endogenous *Lgr4* and the tracing *LacZ*-containing cassette may also result in different effects on post-transcriptional or translational regulation.

Our findings indicating the presence of a *Lgr4* splice variant encoding only the Lgr4 ectodomain is novel for the group B LGRs. Although it is well known that splicing errors may happen in the posttranscriptional processing of premRNAs derived from complex genes, these erroneous mRNAs, which encode potentially toxic polypeptide fragments, are generally eliminated in the cytoplasm rapidly; this will lead to undetectable levels of the corresponding proteins [21]. Our findings showing the consistent presence of the Lgr4-ED protein signal in mouse, rat and bovine gonads strongly suggest this *Lgr4* transcript variant is unlikely to be due to a random splicing error (Fig. 2). Therefore, this naturally occurring *Lgr4* splice variant seems to be highly conserved across a range of mammalian species and thus the translated product may play important, but as yet uncharacterized, roles in modulation of the Lgr4 signaling.

Indeed, alternative splicing of G protein-coupled receptor (GPCR) genes has been demonstrated to be involved in the signal modulation of physiological processes and diseases. For GPCRs with a relatively large N-terminal ectodomain, alternative splicing may generate truncated receptors lacking the transmembrane region and these then serve as dominant-negative antagonists to full-length receptors. Not only found in our study of *Lgr4*, alternative splice variants encoding only the extracellular region of GPCRs have also been reported for several members of the LGR family. For example, in the group A LGRs, a truncated soluble luteinizing hormone receptor lacking the transmembrane region and a putative soluble fragment of follicle-stimulating hormone receptor have been isolated from the turkey ovary and the ovine testis, respectively [22], [23]. Furthermore, a human thyroid-stimulating hormone receptor mRNA variant encoding only the extracellular ligand-binding domain has also been found and reported to play a potential role in thyroid physiology and/or autoimmune thyroid disease [24]. Among the group C LGRs, an alternative *Lgr7* transcript in rodents that encodes a secreted protein containing the low-density lipoprotein class A module has been identified. Expression of this truncated fragment significantly decreases the relaxin-induced signaling of Lgr7 [25]. Beyond the LGR family, splice variants containing only the extracellular region of receptors have also been reported for other GPCRs that have a large ectodomain, such as corticotropin-releasing hormone receptor [26], metabotropic glutamate receptors [27], [28] and gamma-aminobutyric acid B receptor [29]. In the majority of these cases, the truncated proteins seem to act as molecules that are able to bind but are unable to bring about signaling; this allows fine-tuning of the full-length receptor signals.

Lgr4 has been reported to bind R-spondins or norrin; thereby, the forming complex can further enhance the Wnt signaling [3]–[5]. Using reporter assays, we demonstrated that the recombinant Lgr4-ED can indeed dampen the Wnt/β-catenin signaling *in vitro* (Fig. 3). Interestingly, balance of the Wnt/β-catenin signaling has been demonstrated to be crucial for normal development of the male and female reproductive systems. For examples, deficiency in the Wnt signaling, including in Wnt4, Wnt5a, and Wnt7a, will result in severe malformation of the genitals and infertility [30]–[32], whereas hyperactivation of the Wnt/β-catenin pathway can also lead to germ cell apoptosis and male infertility [33], [34]. Thus, the endogenous expression of Lgr4-ED may act as a decoy molecule that aids modulation of the strength of the Wnt/β-catenin signaling in order to maintain appropriate development conditions for the gonads.

*Lgr4*-null mice show strong dilation of the *rete testis* and efferent ducts due to defects in liquid reabsorption. These phenotypes are accompanied by down-regulation of steroid receptors, water transporters and ion transporters, including estrogen receptor, androgen receptor, aquaporin 1, aquaporin 9, Na^+^-K^+^-ATPase and sodium/hydrogen exchanger 3 [17], [35]. Of interest, not only showing the antagonizing effect against the Wnt/β-catenin signaling *in vitro* (Fig. 3), injection of the recombinant Lgr4-ED into mice can also down-regulate the expression of *Esr1* and *Aqp1* in the testis *in vivo* (Fig. 6). Although there is still no consensus regarding the testicular expression and localization of Esr1 in different species and previous studies on *Lgr4*-null mice also indicated the reduction of Esr1 immunostaining was mainly observed in the epididymis and efferent ducts but not in the testis, several recent reports have clearly demonstrated that both mRNA and corresponding protein of *Esr1* can be detected in the Sertoli cells in mouse and rat testes [36]–[38]. In addition, the *Esr1*-knockut male mice are sterile and show atrophic testes with a loss of germ cells in the dilated seminiferous tubules [39], consistent with the phenotypes observed in *Lgr4*-knock male mice [17], [20]. Therefore, the testicular effects of Lgr4 on controlling the *Esr1* expression might potentially explain the consequence of infertility in *Lgr4*-null mice. In addition, although expression of *Aqp1* has been reported to be abundant in the epididymis, several studies have also demonstrated it can be detected locally in some specific cells of the testis. For example, the Aqp1 protein signal can be detected in the endothelium of the blood vessels in the testicular interstitium of cats [40]. Similar results have also been reported in monkeys, rats and mice [41], [42]. These might explain why we can observe the expressional change of *Aqp1* in Lgr4-ED treated mice. Taken together, these findings suggest that Lgr4-ED will be useful as a tool when exploring the possible physiological functions of Lgr4 in various organs.

Taking advantage of this possibility, we used Lgr4-ED to study the potential roles of Lgr4 in the ovary. Although *Lgr4*-null female mice also show significant reduction in fertility [35], no obvious histological change has ever been reported in the ovary. By using superovulated female rats for the Lgr4-ED injection, we showed that Lgr4-ED had suppressive effects on the PMSG induction of *Lhr* expression and on progesterone production in the ovary (Fig. 7). However, we also noticed that changes of steroidogenic gene levels in Lgr4-ED treated animals are not consistent with the dramatic down-regulation of progesterone production. This might be explained by the following reasons. It has been known that the levels of steroidogenic genes can be elevated immediately in the gonadotropin-primed superovulated rodents but then gradually decrease along with the time. Taking *Star* as an example, the *Star* transcript in the superovulated rats has been reported to reach the highest level at 6 hr after PMSG injection and return to almost the basal level after two days [43]. However, for clearly comparing the difference of progesterone levels between control and treatment groups, we designed to harvest the ovaries at 48 hr after treatment to provide enough time for progesterone production and accumulation. This may lead us to miss the best time point for quantification of these genes. In addition, down-regulation of *Lhr*, steroidogenic genes and other yet uncharacterized candidates in Lgr4-ED treated animals may also cause synergetic effects on suppression of progesterone production.

Taken together, our findings suggest that the Lgr4 signaling may help to accelerate the ovarian luteinization process. However, there is a need to consider the possibility that the lack of apparent phenotypes in the *Lgr4*-null mouse ovaries may be due to signal compensation by other ovary-expressed receptors such as Lgr5, Lgr6, and frizzled, which are also involved in similar downstream to activate the Wnt/β-catenin signaling as Lgr4. One possibility to explain our data is that administration of the recombinant Lgr4-ED protein may absorb all the potential Wnt activators, such as R-spondins and norrin, and thus there will be a magnification of Lgr4-ED’s ovarian effects because of the interruption of other potential Wnt/β-catenin signaling driven by other group B LGRs and frizzled proteins. This hypothesis suggests that there is a need to characterize the detailed expression profiles of all the group B LGR members and to identify their ligand signatures across the various ovarian compartments; this will greatly help our understanding of their interplay and relationships.
